# Enterovirus Neutralizing Antibodies, Monocyte Toll Like Receptors Expression and Interleukin Profiles Are Similar Between Non-affected and Affected Siblings From Long-Term Discordant Type 1 Diabetes Multiplex-Sib Families: The Importance of HLA Background

**DOI:** 10.3389/fendo.2020.555685

**Published:** 2020-09-23

**Authors:** Carla Sanchez Bergamin, Elizabeth Pérez-Hurtado, Luanda Oliveira, Monica Gabbay, Valdecira Piveta, Célia Bittencourt, Denise Russo, Rita de Cássia Carmona, Maria Sato, Sergio A. Dib

**Affiliations:** ^1^Endocrinology Division, Department of Medicine, Diabetes Center, Escola Paulista de Medicina - Universidade Federal de São Paulo, São Paulo, Brazil; ^2^Immunology Division, Microbiology, Immunology and Parasitological Department, Escola Paulista de Medicina - Universidade Federal de São Paulo, São Paulo, Brazil; ^3^Laboratory of Dermatology and Immunodeficiencies, LIM-56, Department of Dermatology and Tropical Medicine Institute of São Paulo, Faculdade de Medicina - Universidade de São Paulo, São Paulo, Brazil; ^4^Enteric Diseases Laboratory, Virology Center From Instituto Adolfo Lutz, São Paulo, Brazil

**Keywords:** type 1 diabetes, HLA class II, coxsackievirus, innate immunity, toll-like receptors, islet-cell autoimmunity, multiplex families

## Abstract

Enteroviruses are main candidates among environmental agents in the development of type 1 diabetes (T1D). However, the relationship between virus and the immune system response during T1D pathogenesis is heterogeneous. This is an interesting paradigm and the search for answers would help to highlight the role of viral infection in the etiology of T1D. The current data is a cross-sectional study of affected and non-affected siblings from T1D multiplex-sib families to analyze associations among T1D, genetic, islet autoantibodies and markers of innate immunity. We evaluated the prevalence of anti-virus antibodies (Coxsackie B and Echo) and its relationships with human leukocyte antigen (HLA) class II alleles, TLR expression (monocytes), serum cytokine profile and islet β cell autoantibodies in 51 individuals (40 T1D and 11 non-affected siblings) from 20 T1D multiplex-sib families and 54 healthy control subjects. The viral antibody profiles were similar among all groups, except for antibodies against CVB2, which were more prevalent in the non-affected siblings. TLR4 expression was higher in the T1D multiplex-sib family's members than in the control subjects. TLR4 expression showed a positive correlation with CBV2 antibody prevalence (*r*S: 0.45; *P* = 0.03), CXCL8 (*r*S: 0.65, *P* = 0.002) and TNF-α (*r*S: 0.5, *P* = 0.01) serum levels in both groups of T1D multiplex-sib family. Furthermore, within these families, there was a positive correlation between HLA class II alleles associated with high risk for T1D and insulinoma-associated protein 2 autoantibody (IA-2A) positivity (odds ratio: 38.8; *P* = 0.021). However, the HLA protective haplotypes against T1D prevalence was higher in the non-affected than the affected siblings. This study shows that although the prevalence of viral infection is similar among healthy individuals and members from the T1D multiplex-sib families, the innate immune response is higher in the affected and in the non-affected siblings from these families than in the healthy controls. However, autoimmunity against β-islet cells and an absence of protective HLA alleles were only observed in the T1D multiplex-sib members with clinical disease, supporting the importance of the genetic background in the development of T1D and heterogeneity of the interaction between environmental factors and disease pathogenesis despite the high genetic diversity of the Brazilian population.

## Introduction

Complex interactions between genetic, environmental factors and immune-mediated mechanism are components of type 1 diabetes mellitus (T1D) pathogenesis. Over time and with repeated environmental exposures, an imbalance occurs in the immunological system, triggering an autoimmune response against pancreatic β-cells that results in the progressive destruction of these cells, resulting in insulin deficiency and hyperglycemia (1).

The incidence of T1D has increased worldwide, and between 2005 and 2020, it is predicted that the number of new cases in children younger than 5 years in Europe will double. The prevalence of cases in people younger than 15 years will also increase by 70% in this period (2). In our country (Brazil), the incidence of T1D, in individuals <14 years old, has increased 9.6 times in the last two decades (3, 4). Nevertheless, the incidence of T1D in adults is also increasing worldwide (4).

Nevertheless, in recent years, a decrease in the prevalence of high-risk genotypes and a concomitant increase in the prevalence of protective haplotypes have been observed (5). The HLA (human leukocyte antigen) DRB1^*^03/DRB1^*^04 genotype is as prevalent in Brazilian T1D individuals as in other Caucasian T1D populations despite the high genetic and ethnic diversity (6).

The increase in the prevalence of T1D and the decrease in the prevalence of genetic risk to the disease may indicate that environmental factors could play an important role in this global rise in the incidence of T1D worldwide (7).

Environmental factors may initiate and possibly sustain, accelerate or retard damage to β-cells (7–9). Viruses, particularly enteroviruses, have been identified as causative agents of T1D, as demonstrated in recent clinical studies of enterovirus infection and autoimmunity/T1D (10–12). However, the mechanisms that underlie the onset of T1D remain poorly understood.

Efficient recognition of an infecting virus is imperative for generating a robust immune response during an acute viral infection (13). The initial defense against infectious agents is mediated by receptors known as Toll-like receptors (TLRs), which recognize conserved molecular patterns on different organisms. TLR signaling can up-regulate the expression of co-stimulatory molecules and pro-inflammatory cytokines and induce chemokine production, thereby activating both innate and acquired immune responses. TLR signaling is of particular interest with respect to enterovirus infection because these viruses can activate a strong innate immune response, which has been implicated in T1D pathogenesis (14–17).

A weak innate immune response may allow unrestricted viral replication and the systemic spread of virus. A robust response limits viral replication but may trigger the activation of self-reactive T cells (18, 19). Although considered highly cytolytic, enteroviruses can still persist in various tissues. In genetically predisposed individuals, this persistence may trigger autoimmune-mediated destruction of β-cells, mainly through chronic inflammation (20).

The mechanisms and pathways through which enteroviruses may trigger autoimmune responses and β-cell destruction in genetically susceptible individuals with T1D involve complex interactions among viruses, β-cells and the innate and acquired immune systems which are difficult to model experimentally. These interactions can have different outcomes based on the genetic background of an individual. Two pertinent questions are whether alterations in these pathways in genetically susceptible individuals lead to modifications in innate immune responses to pathogens and whether these potential disturbances are implicated in the pathogenesis of T1D.

Studies of T1D multiplex families whose genetic and environmental factors are relatively less variable may highlight individual characteristics associated with this disease development. The aim of this study was to verify the interrelationship among past enterovirus infection, T1D, genetic, islet autoantibodies and markers of innate immunity in probands with T1D and their non-affected siblings from T1D multiplex-sib families.

## Subjects and Methods

The study population included T1D multiplex-sib families (with at least 2 T1D siblings, but not twins, by family), who were diagnosed according to American Diabetes Association criteria (21, 22). A total of 20 families with 40 T1D patients (2 siblings with T1D in each family), 11 his/her siblings not affected by T1D (T1DNAS) and 54 healthy case-control (N) subjects were included. The study population was recruited from the outpatient clinic of the Diabetes Center of the São Paulo Federal University (São Paulo, SP, Brazil). All the families were from southeastern region of Brazil. Current or recent infections were the exclusion criteria for this study protocol. This study was approved by the local ethics committee of institution (number: CEP 0131/10), and informed consent was signed by all the subjects or their parents.

Each individual underwent a clinical evaluation that included medical history, weight, height and BMI (normal weight: ≥5th and <85th percentile, overweight/obesity ≥85th percentile for children and teens of the same age and sex; normal weight: 18.5 kg/m^2^ and 25 kg/m^2^ and overweight/obesity ≥25 kg/m^2^ for adults). Serum samples were collected from a peripheral vein of healthy subjects and patients for laboratory evaluations and enterovirus neutralizing antibody analysis. TLRs were measured in heparin samples, while serum was taken for detection of interleukins and assessment of islet cells autoantibodies. Monocyte TLR expression was measured in a subset of samples that was representative of all individuals studied [control subjects (*N* = 17), T1D patients from multiplex families (*N* = 17) and their non-affected siblings (*N* = 6)]. All of them [T1D (*N* = 40) and T1DNAS (*N* = 11)] from the T1D multiplex families underwent HLA genotyping.

Samples were taken at 5-year follow-up of non-affected siblings from the T1D multiplex families (*N* = 10) for laboratory.

### Laboratory Evaluation

Complete blood cell counts were determined using an automated chemistry analyzer (Advia 120; Siemens, Germany), and fasting plasma glucose (FPG) levels were determined using glucose hexokinase II (Advia 1650; Bayer, Germany). GHb levels were measured in whole blood using high-performance liquid chromatography (Tosoh Bioscience, USA; normal value 3.5–5.6%). Serum C-peptide levels were measured using an immunofluorometric assay (Auto Delfia, Finland) with a detection limit of 0.10 ng/mL (to convert to nanomoles per liter, multiply by 0.331). The intra-assay variation was 4.2% (value: 0.52 ng/mL), and the inter-assay variations were 1.1% (0.52 ng/mL) and 3.4% (6.1 ng/mL). Serum thyroid-stimulating hormone (TSH) levels were measured using a chemiluminescence assay (Advia Centaur, Bayer, USA; normal value: 0.3–5.0 mUI/ mL).

### Enterovirus Antibodies

Neutralizing enterovirus antibodies were measured against a standard enterovirus panel using a micro-neutralization assay at the Adolfo Lutz Institute, Virology Center, Enteric Diseases Laboratory, São Paulo, SP, Brazil. Sera were diluted 2-fold, from 1:8 to 1:1024, in triplicate, and each dilution was incubated for 2 h at 37°C with 100 TCD 50/0.1 mL of a cell culture infectious dose of the standard antigens from six serotypes of coxsackievirus (CBV1, CBV2, CBV3, CBV4, CBV5, and CBV6) and three serotypes of echovirus (E6, E7, and E30). The virus-serum mixtures were added to RD cells (human rhabdomyosarcoma, ATCC-CCL-136), and after a 72-h incubation at 37°C, the cytopathic effect was assessed using phase contrast microscopy. Titers >1:8 were defined as indicative of protected immunity, and the data were analyzed using Epi Info version 3.4.3 (CDC, Atlanta, GA, USA).

### Innate Immunity

TLR expression in monocytes: Peripheral blood mononuclear cells were isolated from heparinized blood samples by Ficoll-Hypaque gradient centrifugation (GE Healthcare Bio-Sciences AB, Uppsala, Sweden). The cells were then incubated for 20 min at 4°C with an anti-CD32 antibody to block Fc receptors. After washing, the cells were labeled for 1 h at 4°C with fluorochrome-conjugated monoclonal anti-human antibodies (BD Pharmingen, CA, USA) to identify monocytes (based on CD14 expression) and measure TLR expression levels (TLR2, TLR3, TLR4, and TLR9). The cells were then washed and analyzed in a FACS Canto flow cytometer (BD Bioscience, CA, USA) using the FACS-Diva (BD Bioscience) and Flow Jo 10.0.6 (Tree Star, Ashland, OR, USA) software programs. The percentages of positive cells were converted into absolute numbers (cells/mL) based on the total monocyte concentrations obtained through automated cell counting (Advia 120; Siemens, Germany). TLR expression levels were determined through the mean fluorescence intensity (MFI) and were analyzed individually for each population.

Serum interleukin detection: The expression levels of cytokines (IL-1β, IL-6, IL-10, and TNF-α) and chemokines (CXCL8, CXCL9, CCL2, CCL5, and CXCL10) from undiluted patient serum were determined using cytometric bead array kits (BD Biosciences, CA, USA) and analyzed by flow cytometry on a LSR Fortess a cell analyzer (BD Biosciences). The assay sensitivities were as follows: IL-1β (value: 48.4 fg/mL), IL-6 (68.4 fg/mL), IL-10 (13.7 fg/mL), TNF-α (67.3 fg/mL), CXCL8 (0.2 pg/mL), CXCL9 (2.5 pg/mL), CCL2 (2.7 pg/mL), CCL5 (1.0 pg/mL), and CXCL10 (2.8 pg/mL).

### Islet β-Cell Autoantibodies

Glutamic acid decarboxylase autoantibodies (GADA) and insulinoma-associated protein 2 autoantibodies (IA-2A) were analyzed with a commercial radioimmunoassay kit (RSR Limited, UK); we defined positive titers as >1.72 and >0.97 U/mL, respectively (23).

### HLA Genotyping

HLA typing was performed on samples derived from 40 T1D patients and 11 T1DNAS from the multiplex T1D families. DNA was extracted from peripheral blood using a conventional salting out procedure. HLA class II genotyping was performed by a reverse sequence-specific oligonucleotide (SSO) DNA typing method using Luminex technology (LAB Type® SSO Typing Tests-One Lambda, INC., Canoga Park, CA, USA), and low-resolution DRB1 and high-resolution DQA1 and DQB1 were used to evaluate the haplotypes/genotypes related as risk factors for or protective factors against T1D. The following genotypes were considered risk factors for Brazilian T1D: DRB1^*^03/DRB1^*^04, DRB1^*^03/DRB1^*^03 and DRB1^*^04/DRB1^*^04. The following haplotypes were considered risk factors for T1D: DRB1^*^03-DQA1^*^05:01-DQB1^*^02:01, DRB1^*^04-DQA1^*^03:01-DQB1^*^03:02 and DRB1^*^04-DQA1^*^03:03-DQB1^*^03:02. Finally, the following haplotypes were considered protective against Brazilian T1D: DRB1^*^01-DQA1^*^01:01-DQB1^*^05:01, DRB1^*^07-DQA1^*^02:01-DQB1^*^02:02, DRB1^*^08-DQA1^*^04:01-DQB1^*^04:02, DRB1^*^11-DQA1^*^05-DQB1^*^03:01, DRB1^*^13-DQA1^*^01:02-DQB1^*^06:04, DRB1^*^13-DQA1^*^01:03-DQB1*06:02, DRB1*13-DQA1*05:01-DQB1^*^03:01 and DRB1^*^15-DQA1^*^01:02-DQB1^*^06:02 (6).

### Statistical Analysis

Continuous variables were tested for normality of distribution with the Kolmogorov-Smirnov and expressed as mean ± standard deviation or median as appropriate. Categorical variables are presented in tables as absolute numbers (n) and relative (%) frequencies. For these variables, differences between groups were analyzed using chi-square tests, and Fisher's exact test was performed if an expected value was <5. Continuous variables are expressed as medians and interquartile ranges. For these variables, differences between groups were analyzed using the Kruskall-Wallis test, and when the Kruskall-Wallis test was significant, a Konietschke multiple comparison was performed (24). Spearman's rank correlation was computed to assess associations between variables. Multivariate linear regression analyses were performed to identify variables that were independently related to autoimmunity. Statistical significance was set at *P* ≤ 0.05. Data analysis was conducted using commercial software (SPSS version 19 for Windows, SPSS Inc.).

## Results

There were no significant differences among the control, T1D and T1DNAS groups in relation to age, gender or BMI (Table 1). Fasting serum C-peptide levels were similar between the control and T1DNAS groups.

**Table 1:** Clinical, metabolic and endocrine characterization of control subjects, type 1 diabetes (T1D) patients from multiplex families and their non-affected siblings (T1DNAS) studied.

	**Control**	**T1D**	**T1DNAS**	*P*-value**
Number	54	40	11	
Age (yr) (median and range)	18 (14–24)	18 (13–22)	22 (17–27)	Control vs. T1D: ns Control vs. T1DNAS: ns T1D vs. T1DNAS: ns
Male, *n* (%)	23 (43)	24 (60)	7 (64)	Control vs. T1D: ns Control vs. T1DNAS: ns T1D vs. T1DNAS: ns
Duration of T1D (yr)	–	7 (0.1–18)	–	–
**BMI**				
Normal (%)	59	68	46	Control vs. T1D: ns Control vs. T1DNAS: ns T1D vs. T1DNAS: ns
Overweigh/obesity (%)	41	32	54	Control vs. T1D: ns Control vs. T1DNAS: ns T1D vs. T1DNAS: ns
FPG (mg/dL)	89 (83–92)	121 (84–275)	86 (80–90)	Control vs. T1D: <0.001 Control vs. T1DNAS: ns T1D vs. T1DNAS: <0.001
GHb (%) (mmol/mol)	5.6 (5.4–5.8) 38 (36–40)	9.6 (8.2–11.5) 81 (66–102)	5.5 (5.1–5.8) 37 (32–40)	Control vs. T1D: <0.001 Control vs. T1DNAS: ns T1D vs. T1DNAS: <0.001
FCP (ng/mL)	1.64 (1.03–2.44)	0.1 (0.1–0.28)	1.08 (0.34–1.82)	Control vs. T1D: <0.001 Control vs. T1DNAS: ns T1D vs. T1DNAS: <0.001
TSH (μUI/mL)	1.9 (1.38–2.68)	1.9 (1.2–3.2)	1.7 (1.2–3.4)	Control vs. T1D: ns Control vs. T1DNAS: ns T1D vs. T1DNAS: ns

*Data are expressed as median and interquartile range or n (%); T1D, type 1 diabetes; T1DNAS, type 1 diabetes non-affected siblings; FPG, fasting plasma glucose; FCP, fasting C-peptide; TSH, serum thyroid-stimulating hormone; NS, not significant*;

** *Kruskal-Wallis test, and when the Kruskal-Wallis test was significant, a Konietschke multiple comparison was performed*.

The prevalence of CBV1 and CBV6 subtypes were lower than other enteroviruses studied. The only neutralizing enterovirus antibodies whose prevalence differed between the controls and the individuals from the T1D multiplex families were antibodies against CBV2, which were more prevalent in the T1DNAS group than the control group (Table 2).

**Table 2:** Neutralizing antibodies* against different enteroviruses serotypes in control subjects, type 1 diabetes (T1D) patients from multiplex families and their non-affected siblings (T1DNAS).

**Virus neutralizing antibodies**	**Control *N* = 54**	**T1D *N* = 38**	**T1DNAS *N* = 11**	*P*-value**
CBV1 Median antibody title (range)^#^ Antibody prevalence (%)	4 (4–10) 33.3	4 (4–8) 31.6	4 (4–8) 36.4	Control vs. T1D: ns Control vs.T1DNAS: ns T1D vs. T1DNAS: ns
CBV2 Median antibody title (range) Antibody prevalence (%)	12 (4–40) 70.4	16 (4-80) 68.4	64 (16–128) 100.0	Control vs. T1D: ns Control vs.T1DNAS: <0.05 T1D vs. T1DNAS: ns
CBV3 Median antibody title (range) Antibody prevalence (%)	8 (4–16) 53.7	16 (4–32) 63.2	8 (4–16) 63.6	Control vs. T1D: ns Control vs.T1DNAS: ns T1D vs. T1DNAS: ns
CBV4 Median antibody title (range) Antibody prevalence (%)	16 (8–64) 85.1	24 (7–128) 76.3	32 (4–64) 72.7	Control vs. T1D: ns Control vs.T1DNAS: ns T1D vs. T1DNAS: ns
CBV5 Median antibody title (range) Antibody prevalence (%)	6 (4–16) 50.0	4 (4–32) 42.1	4 (4–16) 36.3	Control vs. T1D: ns Control vs.T1DNAS: ns T1D vs. T1DNAS: ns
CBV6 Median antibody title (range) Antibody prevalence (%)	4 (4–8) 33.3	4 (4–8) 36.8	4 (4–8) 27.3	Control vs. T1D: ns Control vs.T1DNAS: ns T1D vs. T1DNAS: ns
E-6 Median antibody title (range) Antibody prevalence (%)	4 (4–8) 33.3	4 (4–16) 39.5	4 (4–32) 45.5	Control vs. T1D: ns Control vs.T1DNAS: ns T1D vs. T1DNAS: ns
E-7 Median antibody title (range) Antibody prevalence (%)	4 (4–16) 40.7	4 (4–16) 36.8	4 (4–64) 45.5	Control vs. T1D: ns Control vs.T1DNAS: ns T1D vs. T1DNAS: ns
E-30 Median antibody title (range) Antibody prevalence (%)	8 (4–32) 64.8	8 (4–32) 60.5	8 (4–32) 72.7	Control vs. T1D: ns Control vs. T1DNAS: ns T1D vs. T1DNAS: ns

* *Neutralizing antibody-positive were samples having a titer ≥8 by plaque assay*;

# *Median antibody title (range)*.

*Data were expressed in median and interquartile range; T1D, type 1 diabetes; T1DNAS, type 1 diabetes non-affected siblings; CBV, group B coxsackievirus; E, echovirus; NS, not significant*;

** *Kruskal-Wallis test, and when the Kruskal-Wallis test was significant, a Konietschke multiple comparison was performed*.

**Figure 1:**
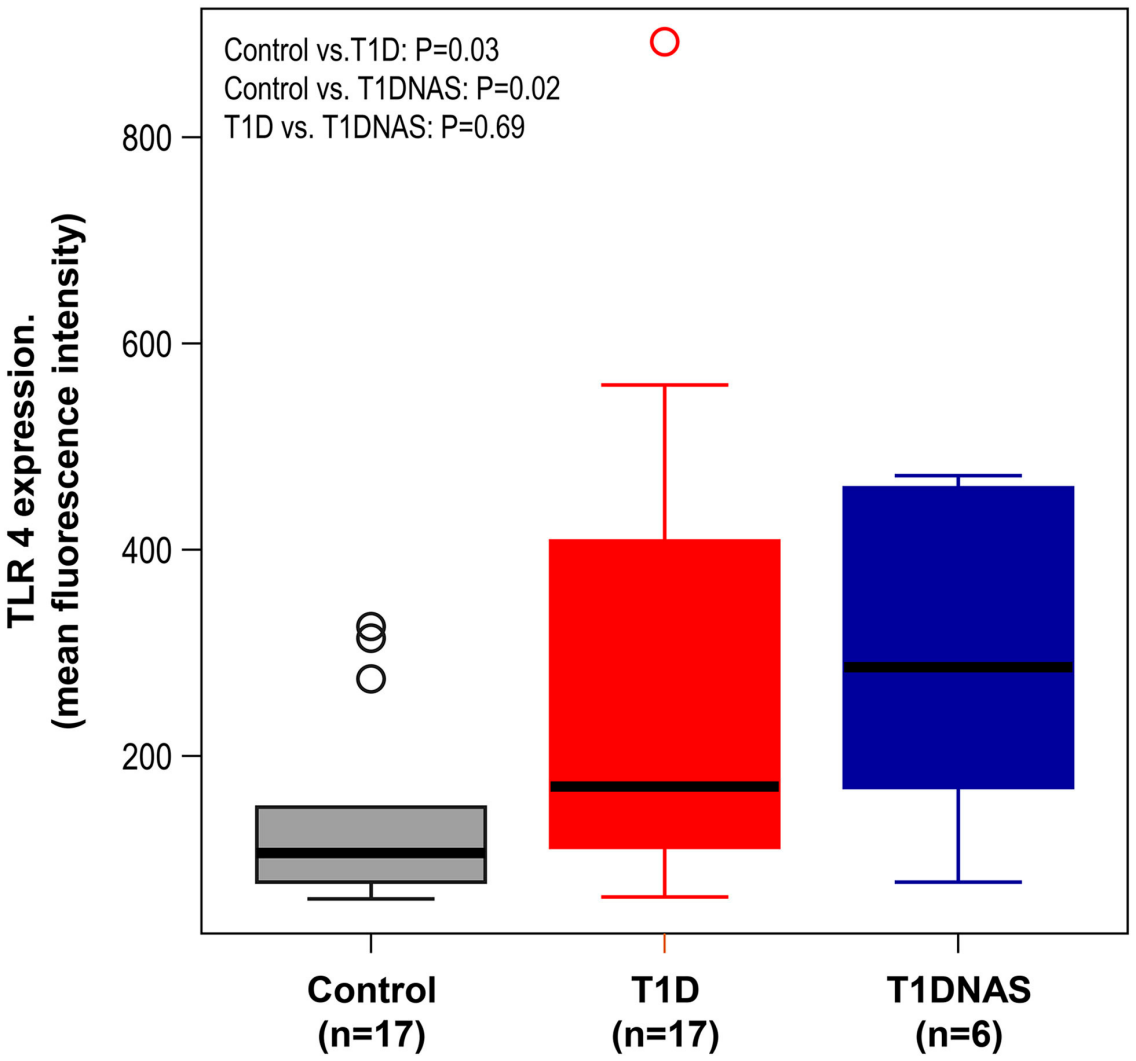
Expression level of TLR4 in monocytes by mean fluorescence intensity (MFI) in control subjects (*N* = 17), type 1 diabetes patients from multiplex families (T1D) (*N* = 17) and their non-affected siblings (T1DNAS) (*N* = 6). Control vs.T1D: *P* = 0.03; Control vs. T1DNAS: *P* = 0.02; T1D vs. T1DNAS: *P* = 0.69.

**Figure 2:**
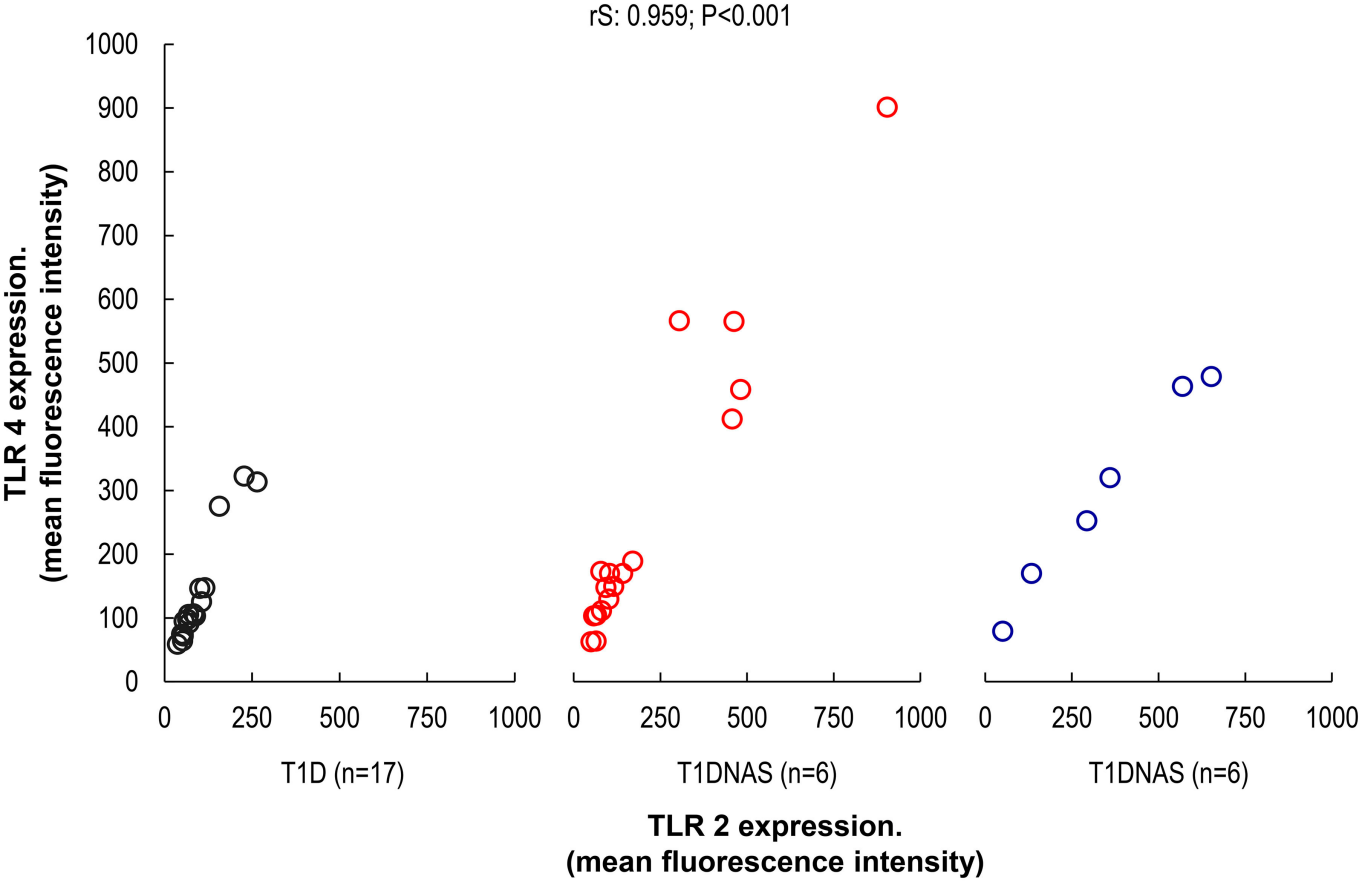
Correlation between TLR2 and TLR4 expression in monocytes by mean fluorescence intensity (MFI) in control subjects (*N* = 17), type 1 diabetes patients (T1D) (*N* = 17) from multiplex families and their non-affected siblings (T1DNAS) (*N* = 6) rS: 0.959; *P* < 0.001.

TLR4 expression in monocytes was higher in the T1D patients and their T1DNAS than in the control subjects (Figure 1). There was a positive correlation between TLR2 and TLR4 expression in monocytes in all three studied groups (Figure 2).

**Figure 3:**
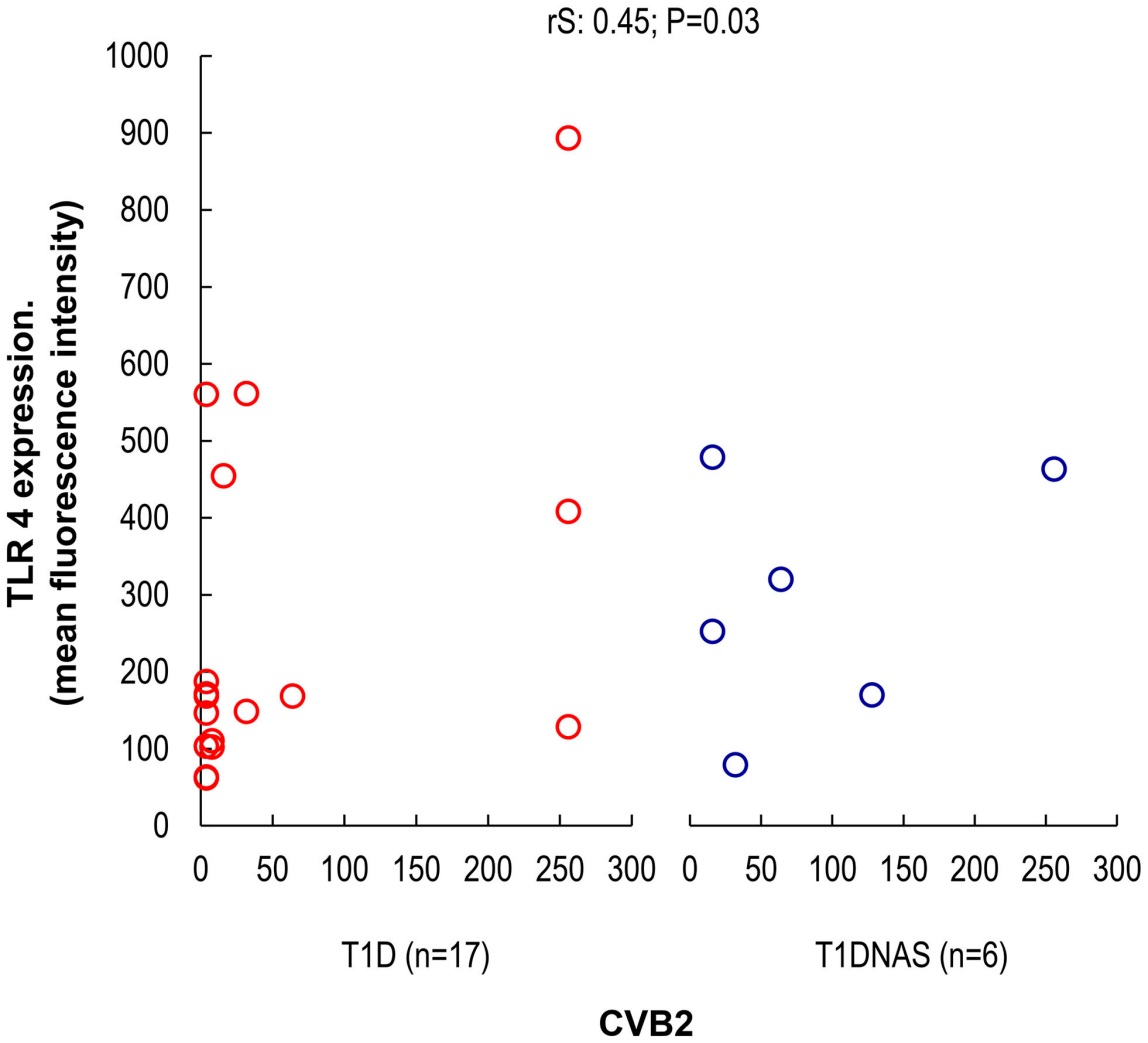
Correlation between TLR4 expression in monocytes by mean fluorescence intensity (MFI) and the presence of neutralizing antibodies against CBV2 in type 1 diabetes patients (T1D) (*N* = 17) and their non-affected siblings (T1DNAS) (*N* = 6) rS: 0.45; *P* = 0.03.

**Figure 4:**
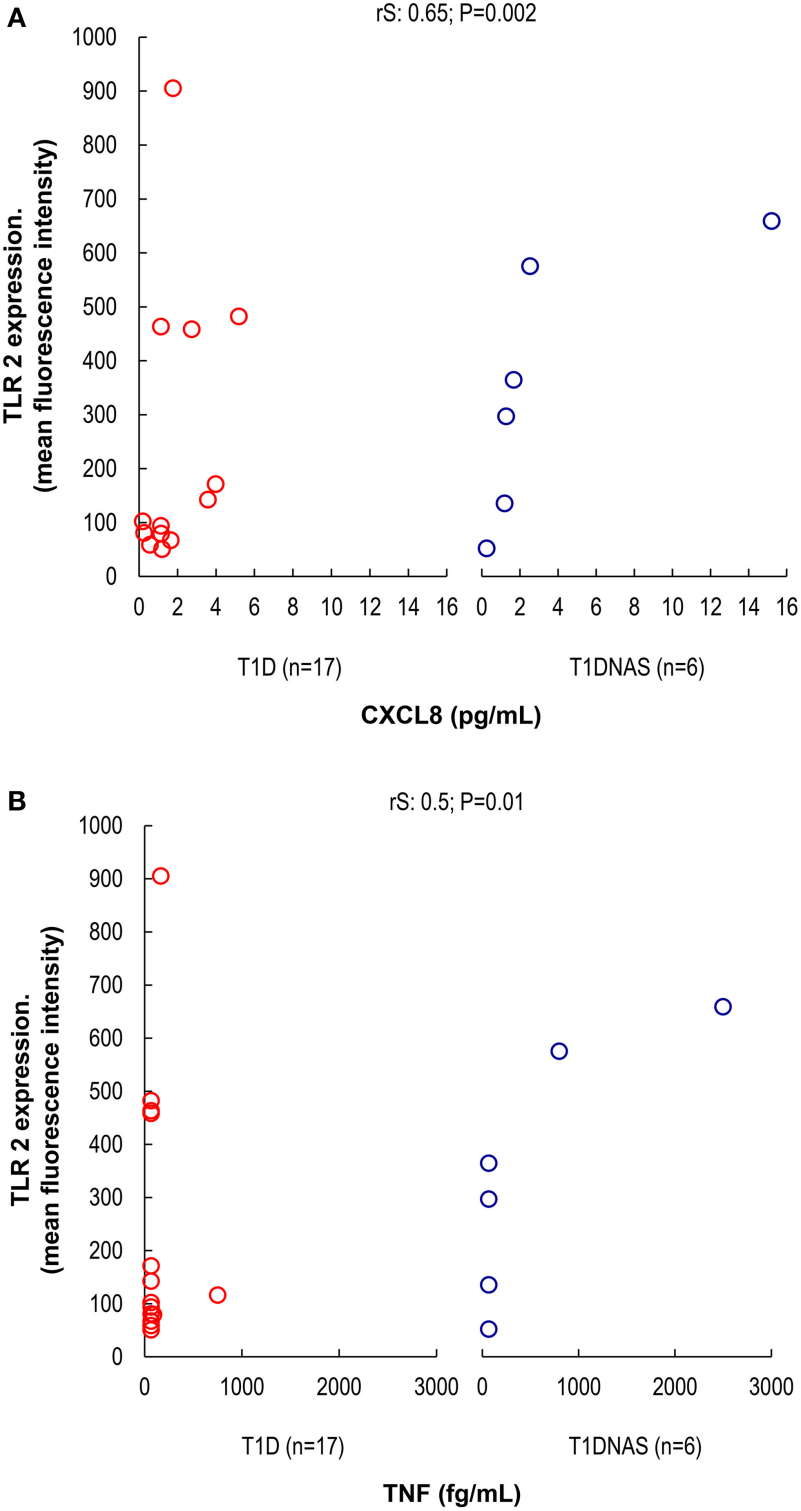
**(A)** Correlation between TLR2 expression and serum concentration of CXCL8 (pg/mL) in monocytes by mean fluorescence intensity (MFI) in patients with type 1 diabetes (T1D) (*N* = 17) and their non-affected siblings (T1DNAS) (*N* = 6) rS: 0.65; *P* = 0.002. **(B)** Correlation between TLR2 expression and serum concentration of TNF-α (fg/mL) in monocytes by mean fluorescence intensity (MFI) in patients with type 1 diabetes (T1D) (*N* = 17) and their non-affected siblings (T1DNAS) (*N* = 6) rS: 0.5; *P* = 0.01.

**Figure 5:**
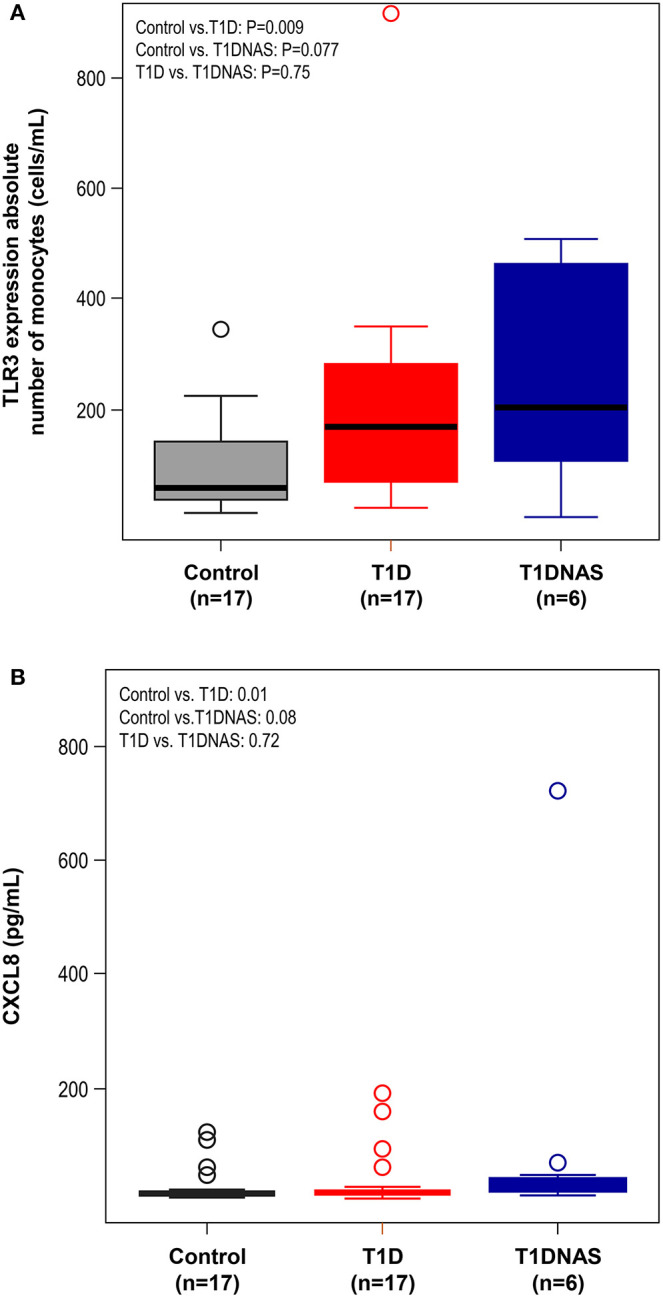
**(A)** Expression level of TLR3 by absolute number of monocytes (cells/mL) in control subjects (*N* = 17), type 1 diabetes patients from multiplex families (T1D) (*N* = 17) and their non-affected siblings (T1DNAS) (*N* = 6). Control vs.T1D: *P* = 0.009; Control vs. T1DNAS: *P* = 0.077; T1D vs. T1DNAS: *P* = 0.75. **(B)** Serum concentration of CXCL8 (pg/mL) in monocytes in control subjects (*N* = 17), type 1 diabetes patients from multiplex families (T1D) (*N* = 17) and their non-affected siblings (T1DNAS) (*N* = 6). Control vs.T1D: *P* = 0.01; Control vs. T1DNAS: *P* = 0.08; T1D vs. T1DNAS: *P* = 0.72.

CXCL8 serum levels were significantly higher in the T1D group than in the control and T1DNAS groups (Table 3). Interestingly, there was a positive correlation between TLR2/TLR4 expression and the presence of neutralizing antibodies against CBV2 (*r*S: 0.45; *P* = 0.03) (Figure 3), the serum concentration of CXCL8 (*r*S: 0.65, *P* = 0.002) (Figure 4A) and the serum concentration of TNF-α (rS: 0.5, *P* = 0.01) (Figure 4B) in the patients with T1D and the T1DNAS group. The absolute number of monocytes expressing TLR3 was also positively correlated with CXCL8 serum concentration in these two groups (*r*S: 0.48, *P* = 0.02) (Figures 5A,B).

**Table 3:** Serum concentration of cytokines and chemokine in control subjects, type 1 diabetes (T1D) patients from multiplex families and their non-affected siblings (T1DNAS).

	**Control *N* = 54**	**T1D *N* = 37**	**T1DNAS *N* = 11**	P value**
IL-1β (fg/mL)	48 (48–136)	48 (48)	48 (48–167)	Control vs. T1D: NS Control vs.T1DNAS: NS T1D vs. T1DNAS: NS
IL-6 (fg/mL)	229 (68–455)	144 (68–298)	160 (108–928)	Control vs. T1D: NS Control vs.T1DNAS: NS T1D vs. T1DNAS: NS
IL-10 (fg/mL)	25 (14–91)	33 (14–106)	76 (14–123)	Control vs. T1D: NS Control vs.T1DNAS: NS T1D vs. T1DNAS: NS
TNF-α (fg/mL)	67 (67–84)	67 (67–94)	67 (67–1649)	Control vs. T1D: NS Control vs.T1DNAS: NS T1D vs. T1DNAS: NS
CXCL10 (pg/mL)	31 (19–54)	22 (13–35)	23 (21–46)	Control vs. T1D: NS Control vs.T1DNAS: NS T1D vs. T1DNAS: NS
CXCL9 (pg/mL)	108 (52–185)	61 (39–131)	95 (40–163)	Control vs. T1D: NS Control vs.T1DNAS: NS T1D vs. T1DNAS: NS
CXCL8 (pg/mL)	0.9 (0.4–1.6)	1.6 (0.9–3.2)	2.2 (1.2–9.4)	Control vs. T1D: 0.01 Control vs.T1DNAS: 0.08 T1D vs. T1DNAS: 0.72
CCL5 (pg/mL)	2,500 (2,500–2,500)	2,500 (2,057–2,500)	2,500 (858–2,500)	Control vs. T1D: NS Control vs.T1DNAS: NS T1D vs. T1DNAS: NS
CCL2 (pg/mL)	24 (16–34)	28 (13–34)	19 (15–47)	Control vs. T1D: NS Control vs.T1DNAS: NS T1D vs. T1DNAS: NS

*Data were expressed as median and interquartile range; T1D, type 1 diabetes; T1DNAS, type 1 diabetes non-affected siblings; NS, not significant*;

** *Kruskal-Wallis test, and when the Kruskal-Wallis test was significant, a Konietschke multiple comparison was perfomed*.

Fifty percent of the patients in the T1D group had positivity to GADA (20 of 40 analyzed) or IA-2A (18 of 33 analyzed) and 11(one third) of this T1D group have positivity for both GADA and IA-2A despite a median disease duration of 7 years. No individual from the T1DNAS group presented positive islet-cell pancreatic antibodies and 5.5% of the controls had positivity to IA-2A.

**Figure 6:**
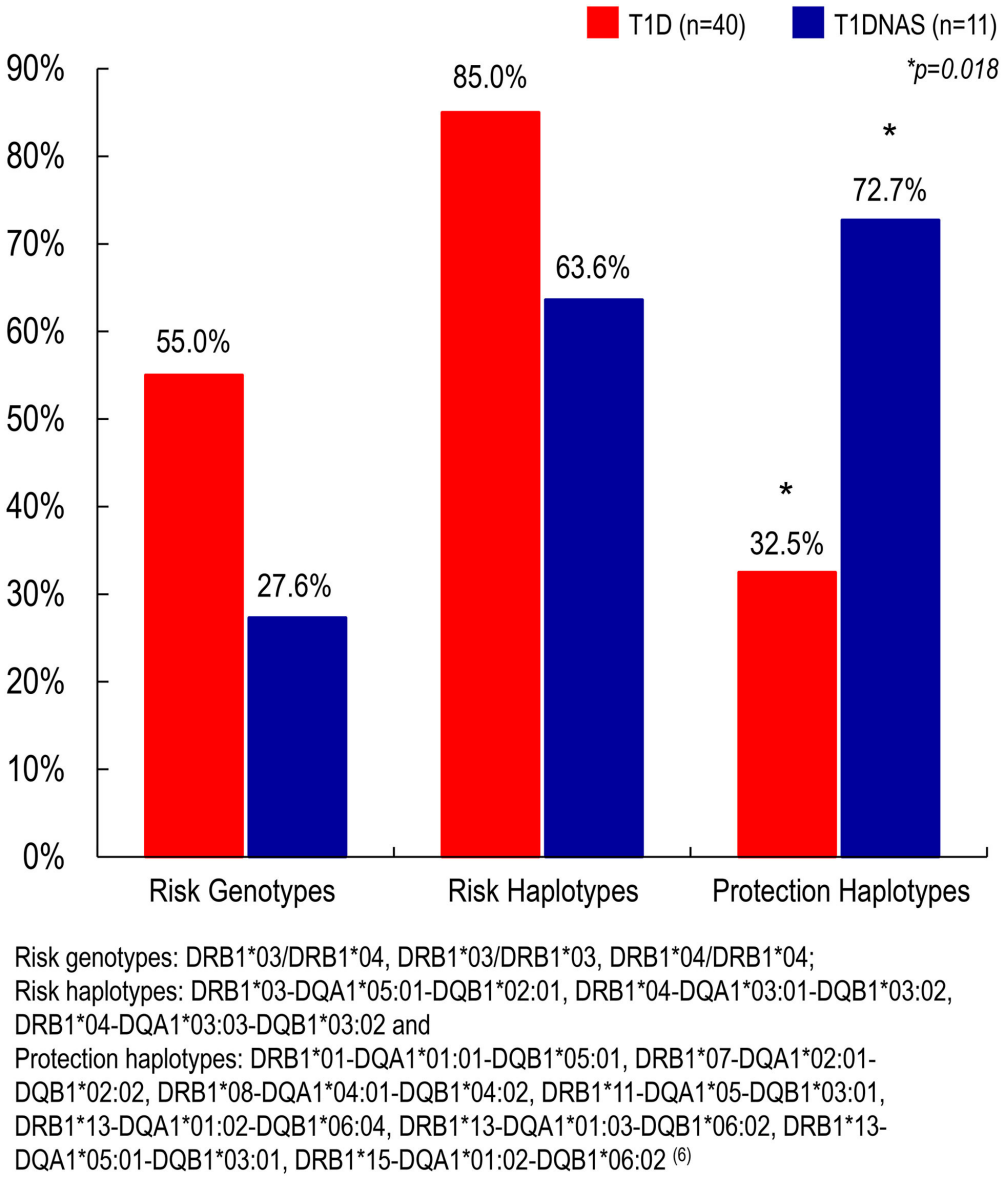
Prevalence of risk genotypes, risk haplotypes and protection haplotypes to type 1 diabetes (T1D) (*N* = 40) in affected and non-affected siblings (T1DNAS) (*N* = 11) of type 1 diabetes multiplex families.

HLA analysis revealed a higher prevalence of protective haplotypes against T1D in the non-affected siblings from the T1D multiplex families than in the affected siblings (Table 4 and Figure 6). Furthermore, there was a correlation between the genotypes identified as risk factors for T1D and IA-2A positivity (odds ratio: 38.8; *P* = 0.021).

**Table 4:** Prevalence of risk genotypes, risk haplotypes, and protection haplotypes to type 1 diabetes (T1D) in affected and non-affected siblings (T1DNAS) of type 1 diabetes multiplex families.

**Prevalence, *n* (%)**	**T1D *N* = 40**	**T1DNAS *N* = 11**	*P*-value**
Risk genotype	22 (55)	3 (27.3)	0.11
Risk haplotype	34 (85)	7 (63.6)	0.122
Protection haplotype	13 (32.5)	8 (72.7)	0.018

*Data were expressed as n and frequency; T1D, type 1 diabetes; T1DNAS, type 1 diabetes non-affected siblings*.

Risk genotypes: DRB1*03/DRB1*04, DRB1*03/DRB1*03, DRB1*04/DRB1*04; risk haplotypes: DRB1*03-DQA1*05:01-DQB1*02:01, DRB1*04-DQA1*03:01-DQB1*03:02, DRB1*04-DQA1* 03:03-DQB1*03:02 and protection haplotypes: DRB1*01-DQA1* 01:01-DQB1*05:01, DRB1*07-DQA1* 02:01-DQB1*02:02, DRB1*08-DQA1* 04:01-DQB1*04:02, DRB1*11-DQA1* 05-DQB1*03:01, DRB1*13-DQA1* 01:02-DQB1*06:04, DRB1*13-DQA1* 01:03-DQB1*06:02, DRB1*13-DQA1* 05:01-DQB1*03:01, DRB1*15-DQA1* 01:02-DQB1*06:02 (6).

** *Chi-square test and Fisher's exact test was performed if an expected value was less than 5*.

Results of a 5-year follow-up of glycemic profile and pancreatic islet autoantibody of the non-affected siblings from the T1D diabetes multiplex families (*N* = 10) revealed that none of these individuals developed T1D and one subject became GADA positive.

## Discussion

The results of the current study showed that affected and non-affected siblings from the multiplex T1D families, except for CBV2, have the same titer of neutralizing virus antibodies as the healthy Brazilian Southwest population analyzed. T1D patients and non-diabetic siblings had similar innate immunity activation (TLR2/TLR4) and inflammatory profiles (CXCL8 and TNF-α), even after a median time period of 7 years from T1D clinical diagnostic in the index cases. However, humoral β-cell autoimmunity was detected only in one non-diabetic sibling during follow-up and was still present in fifty per cent of individuals with a clinical diagnosis T1D. The T1D patients also had a lower prevalence of protective HLA haplotypes to disease than their non-diabetic siblings.

Overall, neutralizing antibodies against CBV are specific markers of past viral infections and our entire population sample studied (controls, T1D and T1DNAS) from the metropolitan Brazil Southwest showed these circulating antibodies. Nevertheless, the prevalence of CBV1 and CBV6 subtypes were lower than other enteroviruses studied. However, CBV2 neutralizing antibody titers were significantly higher in the T1DNAS group than in the control group.

Recently, the role of past exposures to different CBV serotypes was studied by measuring neutralizing antibodies against each of the six serotypes in children newly diagnosed with T1D and in control subjects from five European countries (10). These data were similar to those generated in our study, although our patients with T1D had a >5-year disease duration. In the control group and patients with T1D from Finland, all six CBV serotypes showed similar frequencies of infection based on antibody titers, except for CVB2, which is concordant with our current results. On the other hand, CVB2 antibodies were more prevalent in the control group of the European study and in our data, in the non-affected siblings. Additionally, antibodies against CBV1 were more frequently detected in the children with T1D than in the control group of the European study, suggesting that CBV may include a diabetogenic virus group and that CBV1 may be a member of this group. Indeed, CBV1 was also observed to increase the risk of T1D in the prospective Diabetes Prediction and Prevention (DIPP) study, potentially cause damage to β-cells (25) and might lead to the induction of an autoimmune response against insulin during T1D pathogenesis (26). The capacities of different enteroviruses as risk factors for T1D may vary according to time and place, and different subtypes of coxsackievirus may be implicated in T1D pathogenesis (27, 28). In addition, past reports have indicated that a particular virus can be related to T1D in one population and protective against T1D in another population, evidencing the importance of using serotype-specific methods in such studies (29). In our study, CBV1 antibody prevalence was similar among control, T1D and T1DNAS groups and might not be considered a “diabetic” virus. Therefore, we might speculate that CBV2 is protective against T1D in our studied Brazilian population?

However, it should be noted that enterovirus prevalence varies in different populations and that the vast majority of individuals who become infected with these viruses do not develop diabetes. For example, in a study of Cubans who were exposed to an echovirus epidemic, a large number of patients were found to have seroconverted to β-cell pancreatic autoantibody positivity, but the prevalence of T1D did not increase in these individuals (29).

In principle, the detection of neutralizing antibodies in the patients with T1D from our study covered all past coxsackievirus infections, including coxsackievirus infections that could have occurred prior to, during or after the initiation of β-cell damage. Although the children who developed T1D were likely exposed to the same environmental factors as their unaffected siblings, they did not present the same anti-enterovirus immunity memory for the following possible reasons: (1) overt hyperglycemia may compromise the immune response; (2) we used a cross-sectional retrospective study design, and the prevalence of the neutralizing antibodies that were detected may have underestimated the extent of the link between coxsackievirus infection and disease development; or (3) the “polio hypothesis” held true, suggesting that a previous low number of infections in the T1D population was responsible for reduced protection against the onset of autoimmunity. Indeed, the prevalence of CBV antibodies is lower in Finland than in other European countries, in contrast to the T1D incidence, which is exceptionally high (30). This hypothesis has also been recently supported by experimental studies showing that the absence of maternal enterovirus antibodies increases the risk for severe outcomes following enterovirus infection in offspring (31).

The above-cited studies indicate that the virus-host interactions that occur during the onset of T1D are not acute but rather result from a persistent enterovirus infection followed by cell activation, enhanced expression of adhesion molecules and the production of cytokines of pathogenic significance (20, 32, 33). The relationship between viruses and TLRs is a complex process, involving the modulation of self-reactive T cells (19, 34). In response to pathogenic microorganisms, innate immune response mediated by TLR would result from the mechanism of “bystander activation” (immune phenomenon in which T cells are activated in the absence of T-cell receptor stimulation) in the activation of self-reactive T cells, release of pro inflammatory cytokines resulting in injury to pancreatic β-cells (20, 32–35). During a CBV infection, the activation of the TLR pathway increases the expression of various inflammatory cytokines and chemokines that would activate the pathway of oxidative stress. This process can contribute to pancreatic β cell apoptosis and activation of the acquired immune response against these cells, resulting in the production of autoantibodies and maybe the clinical T1D onset (17, 33). *In vitro* experiments have indicated that CBV can be recognized by TLR4, 7, and 8 (36). Recognition of CBV4 and subsequent cytokine secretion were shown to be dependent upon TLR4 in pancreatic cell lines (36, 37). Studies performed in mouse models of T1D have also implicated TLR1, TLR2, TLR3, and TLR7 in disease mechanisms (37).

The dendritic cells (DCs) are important players against microbial infections and these cells express a different set of TLRs, however the frequencies of the DCs in peripheral blood are extremely low (0.2–0.8%). To overcome this limitation, studies have analyzed innate functions in monocytes whose frequency in the peripheral blood is relatively large (17, 19). Increased expression, activity, and signaling of TLR2 and TLR4 occur more frequently in monocytes from T1D patients compared to those from matched controls (34). Moreover, in the present study, TLR4 expression was higher in affected and non-affected siblings of T1D multiplex families than in the control group. There was a positive correlation between TLR2 and TLR4 expression in their monocytes.

Viral infections trigger the production of pro-inflammatory cytokines, particularly IL-1β and TNF-α, in human pancreatic cells through a TLR4-dependent pathway (18, 37). Additionally, human peripheral blood mononuclear cells experimentally infected with CBV4 have shown enhanced production of cytokines such as TNF-α and IL-6 (38). Although these cytokines appear to have protective roles in host defense against infection, some studies have suggested that these cytokines have a negative effect on pancreatic function, and the involvement of TNF-α in β-cell damage has been reported in the early stages of T1D development by triggering endoplasmic reticulum stress. (39). Actually, IFNα is a key cytokine for a wide range of immune processes. Its induction at the beginning of the innate immune response triggers many mechanisms which will activate both innate and acquired immunity, resulting in stimulation of cytotoxic T cells and beta cell death (34). Accordingly, our data suggest that innate immunity pathways were activated in both the affected and non-affected siblings from the T1D multiplex families because CBV2 serological memory was positively associated with TLR2 and TLR4 expression levels, which were correlated with CXCL8 and TNF-α serum levels in these groups. We can hypothesize that CBV2 infection activates the TLR2, TLR3, and TLR4 pathways, inducing the CXCL8 production by the β-cells with the stimulation of Th1 and Th2 cells to produce TNF-α as has been suggested to CXCL10/CXR3 chemokine system in the autoimmune process (35). Experimental studies showed that TLR3 has an important role in response to a common human viral infection, stimulating CXCL8 (40). We also found in our multiplex T1D families, individuals with a positive correlation between TLR3 and CXCL8.

However, these networks in some situations behave as a double-edge sword and the activation of the process may not result always in the triggering of autoimmunity against pancreatic β-cells. Although patients with T1D and their non-affected siblings may be exposed to the same environmental factors and have similar inflammatory immune responses, in the current study, none of the non-affected siblings were found to have β-cell autoantibodies. In contrast, half of the patients with T1D still presented islet cell autoantibody positivity 7 years after acquiring the disease. As a comparison, the prevalence of β-cell autoantibodies in the T1DNAS group is lower than other Brazilian studies (26, 41). After 5 years of glycemic profile and pancreatic islet autoantibody follow-up of the non-affected siblings from the T1D multiplex families, we found that none of these individuals developed T1D and one subject became GADA positive, but remained normoglycemic.

HLA antigens play a key role in regulating immune responses. Its potential involvement in enterovirus-induced β-cell damage is based on studies demonstrating modulation of the clinical course of infectious diseases such as malaria, hepatitis and HIV by HLA alleles (42–44). High-risk HLA antigens may present β-cell autoantigens to the immune system, leading to an aggressive autoreactive response, whereas protective HLA antigens are unable to induce an aggressive response. However, HLA antigens may regulate immune responses against environmental triggers of T1D, modulating their risk effects (45). A study of a population from Finland indicated that children with HLA-DR risk alleles for T1D do not have defects in their humoral immune responsiveness to enterovirus antigens, which would make these children more susceptible to enterovirus infections. In fact, high-risk HLA-DR alleles were associated with strong immune responsiveness, resulting in exaggerated immune pathology and cell damage in pancreatic islets in enterovirus-infected subjects (46). In a recent study, relatives with protective HLA antigens were less likely to present multiple autoantibodies at baseline, and in follow- up they still had not converted from single to multiple autoantibodies (47). The Brazilian population is characterized by high genetic and ethnic diversity; however, the HLA DRB1^*^03/DRB1^*^04 genotype is as prevalent in Brazilian T1D individuals as observed in other T1D populations (6). Interestingly, in our study, there was a positive correlation between genotype risk for T1D and IA-2A positivity. Specifically, these antibodies appeared to be associated with inflammatory peripheral cytokine/chemokine profiles rather than with GADAs, as shown in another study from our group (48).

The T1DNAS group in the current study did not develop β-cell autoantibodies and presented a higher prevalence of protective HLA alleles than their affected siblings, suggesting that the relationship among antiviral serological memory, TLR expression, interleukin signaling and pancreatic autoimmunity is mediated by genetic factors. As other studies have shown, other candidate genes, such as IFIH1, MDA5, PTPN2, and TYK2 can regulate relationships among viruses, β-cells and the immune system (49, 50).

There are some potential limitations to our study. The non-affected siblings group studied was small and it was not possible to study the monocyte TLR expression in all the participants. Besides this, the data were representative of all populations evaluated. We did not exclude siblings with HLA low-risk genotypes for T1D, who may also have lower risk to develop the disease. We did not investigate the prevalence of zinc transporter 8 autoantibodies (ZnT8A) and insulin autoantibodies (IAA) in non-diabetic siblings. Only glycemic profile and pancreatic autoantibodies were evaluated during the follow-up period in the non-diabetic siblings.

In conclusion, this study in a set of multiplex T1D families corroborates to data showing that the relationships among enterovirus neutralizing antibodies, TLR expression, interleukin profiles and pancreatic β-cell autoimmunity may be related to the individual's genetic background, in particular, to their HLA class II DR and DQ molecules in subjects from Brazilian population. These confirmatory findings support the concept of heterogeneity between environmental factors and T1D development that have already been observed in different populations.

## Data Availability Statement

The raw data supporting the conclusions of this article will be made available by the authors, without undue reservation.

## Ethics Statement

The studies involving human participants were reviewed and approved by Ethics Committee from Universidade Federal de São Paulo (CEP 0131/10). Written informed consent to participate in this study was provided by the participants' legal guardian/next of kin.

## Author Contributions

CB and SD planned the study, the review, and editing of the manuscript. In addition, CB was responsible for recruiting study subjects, performing laboratory work, and drafting the manuscript. MG contributed to study design and subject recruitment. VP and CB helped collect samples. DR and RC organized the analysis of virus neutralizing antibodies. EP-H was responsible for TLR expression analysis. LO and MS were responsible for interleukin expression analysis. SD coordinated the project and was responsible for the scientific management of the project. All authors contributed to the article and approved the submitted version.

## Conflict of Interest

The authors declare that the research was conducted in the absence of any commercial or financial relationships that could be construed as a potential conflict of interest.
